# Interactive and Joint Effects of Obesity and Insulin Resistance on Hypertension in Adolescents and the Mediating Role of Insulin Resistance—Five Provinces, China

**DOI:** 10.3390/nu17172783

**Published:** 2025-08-27

**Authors:** Haiyuan Zhu, Zebang Zhang, Yumei Feng, Qiqi Wu, Runquan Zhang, Tao Liu, Dan Liu, Xiongfei Chen, Xiaomei Dong

**Affiliations:** 1 Department of Public Health and Preventive Medicine, School of Medicine, Jinan University, Guangzhou 510632, China; jnuzhu007@stu2023.jnu.edu.cn (H.Z.); zhangzb117@163.com (Z.Z.); 15573415243@163.com (Y.F.); hestiawu1121@163.com (Q.W.); zhangrunquan3@163.com (R.Z.); gztt_2002@163.com (T.L.); 2 Department of Epidemiology, School of Public Health, Southern Medical University, Guangzhou 510515, China; liudan0717@smu.edu.cn; 3 Department of Primary Public Health, Guangzhou Center for Disease Control and Prevention, Guangzhou 510440, China; chenxf_1975@163.com

**Keywords:** adolescent, China Nutrition and Health Surveillance, hypertension, insulin resistance, obesity, TyG

## Abstract

**Background:** The global prevalence of pediatric hypertension is on the rise. Adolescence is a period of high prevalence of childhood hypertension. Obesity and insulin resistance (IR) are important risk factors in the development of hypertension, but their interaction and combined effects on adolescent hypertension remain unclear. **Methods:** This cross-sectional study utilized data from the China National Nutrition and Health Surveillance of Children and Lactating Mothers (2016–2017). A total of 7031 adolescents aged 12–17 years from five provinces were included. The triglyceride-glucose index (TyG) was used as an indicator of IR. Multivariable logistic regression and generalized linear mixed-effect models were used to assess the interaction and joint effects of IR and obesity (measured by body mass index [BMI] and waist circumference [WC]) on hypertension. Relative excess risk due to interaction (RERI), proportion attributable to interaction (AP), and synergy index (SI) were calculated to evaluate additive interactive effects. Mediation analysis explored the potential mediating role of the TyG in the association between obesity and hypertension. **Results:** IR and obesity were positively associated with adolescent hypertension (*p* < 0.001). Interaction analyses revealed a robust synergistic interaction between obesity and IR on hypertension, with the AP being approximately 40% (*p* < 0.001). TyG significantly mediated the association between obesity and adolescent hypertension (6.30% for high BMI and 8.54% for high WC, both *p* < 0.001). **Conclusions:** This study suggests that obesity and IR could synergistically contribute to the prevalence of hypertension in adolescents. For the primary prevention and management of hypertension in adolescents, strategies targeting both factors should be considered.

## 1. Introduction

Pediatric hypertension is a growing public health concern, with its prevalence increasing in recent decades worldwide [1,2]. A systematic review covering 22,431,861 children reported that in 2020, the overall prevalence of hypertension among Chinese individuals aged 6–18 years was 3.11%, equivalent to 6.80 million affected children [3]. Research shows that the age-specific prevalence rises rapidly from the onset of puberty and peaks at 14–15 years of age [2]. Notably, elevated blood pressure (BP) in childhood can not only induce early cardiovascular damage but also increase the risk of cardiovascular diseases (CVD) and mortality in adulthood [1,4], which highlights the importance of normal BP during childhood and adolescence [4]. Although the BP of minors increases with age, it may rise more during puberty, possibly due to hormonal changes and rapid growth spurts [5,6]. This might also explain why the prevalence of hypertension in children peaks at the end of puberty [2]. Adolescence thus becomes a special period in which individuals are more susceptible to hypertension. Given the long-term health impacts of untreated and unmanaged pediatric hypertension, the importance of early prevention and intervention cannot be overstated.

Obesity is a widely recognized risk factor for hypertension [1,2], particularly abdominal obesity [7]. Body mass index (BMI) and waist circumference (WC) are commonly used to assess the condition of obesity. Obesity can cause fat accumulation in body tissues, which is associated with insulin resistance (IR), a condition characterized by reduced sensitivity and responsiveness to insulin [8]. IR leads to dysfunction in insulin regulation of glucose metabolism [9] and is thought to play a crucial role in the progression and development of hypertension [10,11]. The gold standard for measuring IR, the hyperinsulinemic-euglycemic clamp (HEC) test, is invasive and costly, whereas the triglyceride-glucose index (TyG) is a reliable and cost-effective indicator of IR [10,11] and has been proven to be a useful surrogate marker for IR in adolescents [12]. Obesity and IR are increasingly prevalent metabolic conditions among adolescents, largely driven by modern lifestyle factors such as high intake of sugar and fat and insufficient physical activity [13]. Both conditions could increase the risk of hypertension, and there may be a mechanistic interrelationship between fat accumulation and IR [14].

This study aims to examine the interactive and joint effects of obesity and IR (assessed by TyG) on adolescent hypertension, and to evaluate whether TyG mediates the association between obesity and hypertension among adolescents. To date, no studies have systematically explored the interaction and combined effect of obesity and IR on hypertension from an epidemiological perspective among individuals in adolescence. Considering the potential links and overlaps between obesity and IR, further evaluation into the effects of combined obesity and IR on adolescent hypertension is warranted. The findings may help enhance the understanding of the underlying mechanisms of adolescent hypertension, facilitate risk stratification, and inform targeted interventions for adolescent BP control, reducing their risk of developing CVD in adulthood.

## 2. Methods

### 2.1. Data Sources and Study Population

The data analyzed in the present study came from a nationwide survey, the China Nutrition and Health Surveillance of Children and Lactating Mothers in 2016–2017, which was led by the Chinese National Health Commission (NHC) and conducted by the Chinese Center for Disease Control and Prevention (China CDC), using a multi-stage stratified random sampling. Detailed multi-stage sampling procedures and sample selection processes are available in a published article [15]. In short, for children and adolescents aged 6–17 years, participants were selected from monitoring sites across 31 provincial-level administrative divisions in mainland China, with 280 students recruited at each site. Students with serious physical and mental illnesses did not participate in the program. Subsequently, participants completed face-to-face questionnaires, anthropometric measurements, and provided blood and urine samples for laboratory tests. Eventually, the data collected were nationally and provincially representative.

**Figure 1:**
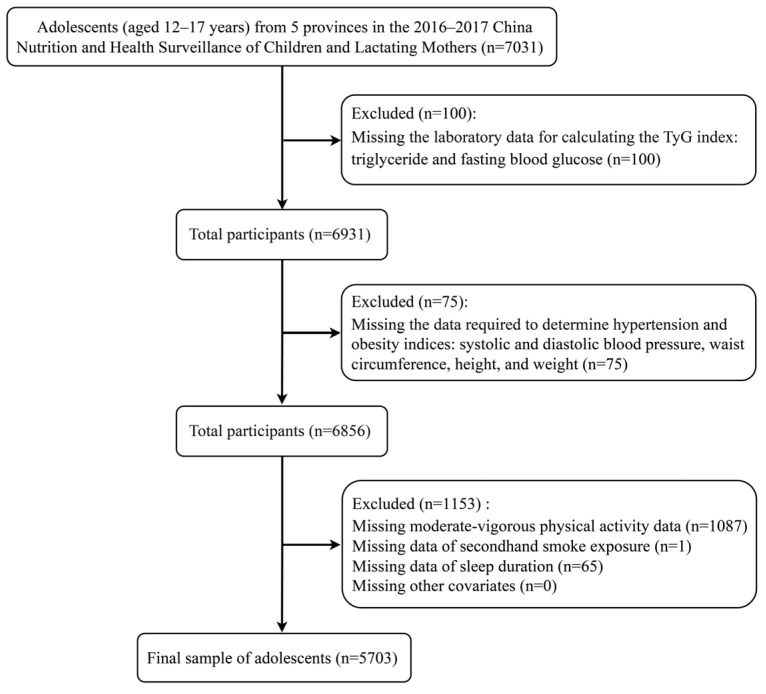
Exclusion flowchart of study participants in our surveillance dataset.

However, the database of this surveillance is not publicly available. We endeavored to seek sectoral cooperation of the relevant authorities to obtain the data and ended up with data for five provinces (Shandong, Jiangsu, Guangdong, Guizhou, and Inner Mongolia). These provinces are located in various regions of China: Northeast, East, South, Southwest, and North. The broad geographic distribution of participants ensured sufficient variability in the study population. Finally, we extracted 7031 adolescents aged 12–17 years from our dataset for our study. However, 1328 participants were excluded due to missing data, as detailed in Figure 1. The specific missingness of data and the details of participants included and not included are shown in Supplementary Materials: Figure S1 and Table S1.

### 2.2. Ethical Considerations

This study received ethical approval from the Ethics Committee of the Chinese Center for Disease Control and Prevention (Protocol Code: 201614). Children and adolescents were included in the program only after their guardians and/or the participants themselves had signed an informed consent form. The study was conducted in accordance with the Declaration of Helsinki.

### 2.3. Assessment of BMI, WC, and TyG

Participants at each survey site were measured using anthropometric equipment selected by the national project team [15] and Chinese health industry standard measurement methods [16]. Height measurements were performed using a TZG-type stadiometer (accuracy up to 0.1 cm) with participants in a standing position having removed their shoes. Body weight was measured to the nearest 0.01 kg using an electronic scale (TANITA HD-390,Tokyo, Japan), with participants wearing only a single layer of clothing and no shoes or outerwear. All equipment was calibrated prior to the physical examination, and measurements were conducted in the morning when participants were in a fasting state. Body mass index (BMI, kg/m^2^) was calculated as weight in kilograms divided by the square of height in meters. Definitions of overweight and obesity in adolescents in this study were based on the standard thresholds of the health industry in China [17]. We classified participants who were overweight or obese as having a high BMI, and otherwise as having a low BMI.

Waist circumference (WC) was measured in the fasting state with participants standing upright. A non-elastic tape was placed horizontally at the midpoint between the lower margin of the costal arch and iliac crest along the mid-axillary line, lightly applied to the skin, and readings were taken at the end of a normal expiration. Two measurements were taken, and both were recorded only if the difference was less than 2 cm; otherwise, the measurement was repeated. In the present study, the average of the two readings was used as the participants’ WC. Abdominal obesity was defined as a WC at or above the 90th percentile for age and sex, determined according to the cut-off points specified in the national health industry criteria of China for children and adolescents [18,19]. We referred to abdominal obesity as high WC, and non-abdominal obesity as low WC.

In order to test their laboratory indicators, including triglyceride (TG) and fasting blood glucose (FG), 6 mL of fasting blood and 8–10 mL of random urine were collected from participants. TG levels were measured via enzymatic colorimetry using the Roche Cobas C701 automated analyzer, and FG was assessed using the glucokinase method with a Roche P800 biochemical analyzer (Roche, Basel, Switzerland). TyG was calculated as ln[TG (mg/dL) × FG (mg/dL)/2] [11], and the median (8.18) was used as the cut-off point in this study, classifying the TyG into ‘low’ and ‘high’ categories. Notably, 8.18 is thought to be the TyG cut-off for diagnosing IR in adolescents [12].

### 2.4. Assessment of Hypertension

BP was measured in the morning using an electronic sphygmomanometer (OMRON HBP-1300, Tokyo, Japan), accurate to 1 mmHg. Participants were instructed to avoid exercise, eating, or drinking (except water) for at least one hour before the measurement. Unless contraindicated, BP measurements were performed on the left arm. A total of three measurements were taken at 1 min intervals. The average of the two closest values among the three BP measurements was used in this study. Hypertension in adolescents was defined as systolic BP and/or diastolic BP ≥ 95th percentile for sex, age, and height, based on the reference of screening for Chinese children and adolescents [20].

### 2.5. Assessment of Covariates

Data on sociodemographic information, including date of birth (month/day/year), sex (male or female), father’s and mother’s education levels (≤primary education, secondary education, or ≥bachelor’s degree), and residence (rural or urban), were collected through a structured questionnaire by interviewing participants face to face. The age used as a covariate was derived from the interval between the date of birth and the date of data collection, rounded down to the nearest whole year. To avoid collinearity, father’s and mother’s education levels were combined into a single variable—parental education level—classified as low (both parents ≤ primary education, or one ≤ primary and the other received secondary education), medium (both secondary, or one ≤ primary and the other ≥ bachelor’s degree), and high (both ≥ bachelor’s, or one secondary and the other ≥ bachelor’s).

Investigators also collected health-related information such as frequency of moderate-vigorous physical activity (MVPA), bedtime and wake-up time, secondhand smoke, alcohol intake, and family history of hypertension using the questionnaire. The frequency of MVPA (continuous) was obtained by asking participants about the number of days they typically engaged in MVPA per week. Participants’ sleep duration was calculated as the interval between their bedtime and wake-up time. By the minimum sleep duration recommendations for children and adolescents (≥8 h/day for high school students, ≥9 h/day for middle school students, and ≥10 h/day for primary school pupils) issued by the Ministry of Education of the People’s Republic of China [21], participants meeting these criteria were considered to have adequate sleep; otherwise, they were considered insufficient. Family history of hypertension was considered present if at least one of the following relatives had been diagnosed with hypertension: father, mother, paternal grandparents, or maternal grandparents.

We also considered the impact of dietary patterns on outcome variable, so we used dietary data in the database obtained from a Food Frequency Questionnaire (FFQ), which surveyed participants on their frequency of consumption of various foods over the past month, to calculate an unhealthy dietary score for each adolescent, representing a potential confounder from different diets. The scoring criteria were based on the Chinese Dietary Guidelines [22], and the detailed scoring process is accessible in our previously published article [23].

Other covariates were laboratory-tested, including serum uric acid, serum total protein, total cholesterol (TC), urine sodium, and serum creatinine. We calculated estimated glomerular filtration rate (eGFR) as a covariate based on values of serum creatinine to consider a confounder of kidney disease, which can independently contribute to hypertension. A formula recommended by the Chinese guideline for early screening of pediatric chronic kidney disease was used: eGFR = K × height (cm) × 88.4/serum creatinine (μmol/L) (children age 2–12 years: K= 0.55; adolescents age > 12 years: K = 0.77 for boys and 0.55 for girls) [24]. In post-modeling diagnostics, the variance inflation factors of covariates in all models of the present study were less than 5, indicating that there was no major collinearity affecting our regression estimates.

### 2.6. Statistical Analysis

All statistical analyses were performed using R software version 4.2.3 (R Foundation, Vienna, Austria). A two-tailed *p* < 0.05 was considered statistically significant. Quantitative data were presented as means and standard deviations (SDs), while qualitative data were described as numbers and percentages. Pearson’s chi-square test, Student’s *t* test, or Wilcoxon rank sum test were used to compare the differences in variables between groups where appropriate.

Multivariable logistic regression models were used to estimate the odds ratios (ORs) and 95% confidence intervals (CIs) of hypertension associated with TyG, BMI, and WC. In Model 1, age and sex were adjusted. Model 2, the fully adjusted model, was adjusted for sex, age, MVPA, unhealthy dietary score, sleep adequacy, secondhand smoke, alcohol intake, family history of hypertension, parental education level, residence, serum uric acid, serum total protein, eGFR, TC, and urine sodium. Furthermore, we performed restricted cubic spline (RCS) regression to explore potential non-linear relationships, with the knots set to 3 for smooth curve fitting.

The ‘interactionR’ package (version 0.1.7) in R software was used for the calculations of additive interaction and to assess the joint effects of IR and obesity on hypertension in the main analysis. Relative excess risk due to interaction (RERI), proportion attributable to interaction (AP), and synergy index (SI) were used to evaluate additive interactive effects. Multiplicative interactions were assessed through likelihood ratio tests, comparing models with and without the interaction term.

We proceeded to conduct mediation analysis to explore whether IR mediates the link between obesity and adolescent hypertension. The analysis was performed using the ‘mediation’ package (version 4.5.0), incorporating linear and logistic regression models to assess the direct and indirect associations. In the process, a nonparametric bootstrap approach was applied, with continuous TyG as a mediator, allowing us to quantify the proportion of the total effect of obesity on hypertension mediated by TyG.

To investigate the potential heterogeneity of interactive and joint effects in individuals with different characteristics, subgroup analyses stratified by age, sex, MVPA, and sleep adequacy were conducted, and the *p* values for interaction of stratification variables with the interaction terms of ‘TyG × high obesity indices’ were simultaneously calculated. Based on the subgroup results, interaction analysis was re-performed in age groups of 12–14 years and 15–17 years, separately.

Furthermore, several sensitivity analyses were conducted to validate the robustness of the main analysis of interaction in this study. First, a generalized linear mixed-effect model (GLMM) with a logit link function was fitted, with the province included as a random intercept to account for geographical clustering and unmeasured contextual factors (e.g., climatic conditions, pollution levels) that may contribute to between-province differences in hypertension risk. Second, the original entire dataset was used (n = 7031). The missing values were renamed ‘unknown’ for qualitative variables alcohol intake, secondhand smoke, MVPA, and sleep adequacy, which were covariates with missing values exceeding 15% (Supplementary Materials: Figure S1). For the variables with less than 3.5% missing values, their missing values were assumed to be randomly missing, and therefore, multiple imputation based on chained equations using baseline characteristics was used for imputation. We created an imputed dataset using the ‘mice’ package (version 3.16.0) to re-examine previous main findings. Finally, E-values were calculated to estimate the potential impact of unmeasured confounders in the association of TyG and obesity indices with adolescent hypertension.

## 3. Results

### 3.1. Characteristics of Adolescents and the Prevalence of Hypertension

Table 1 presents the characteristics of the participants. A total of 5703 adolescents were finally included in the main analysis, of which 47.07% were boys and 52.93% were girls. The mean age was 14.2 years (SD = 1.7 years). Participants with hypertension exhibited significantly higher levels of BMI, WC, serum uric acid, serum total protein, TG, FG, and TyG, compared with those without hypertension, while the frequency of MVPA was higher in the non-hypertension group (all *p* < 0.05). In addition, compared with the non-hypertension group, the hypertension group had a greater proportion of high BMI (37.06% for hypertension vs. 15.82% for non-hypertension, *p* < 0.001) and high WC (30.39% for hypertension vs. 13.08% for non-hypertension, *p* < 0.001). Further, alcohol consumption, family history of hypertension, and province differed significantly between the two groups (all *p* < 0.01).

**Figure 2:**
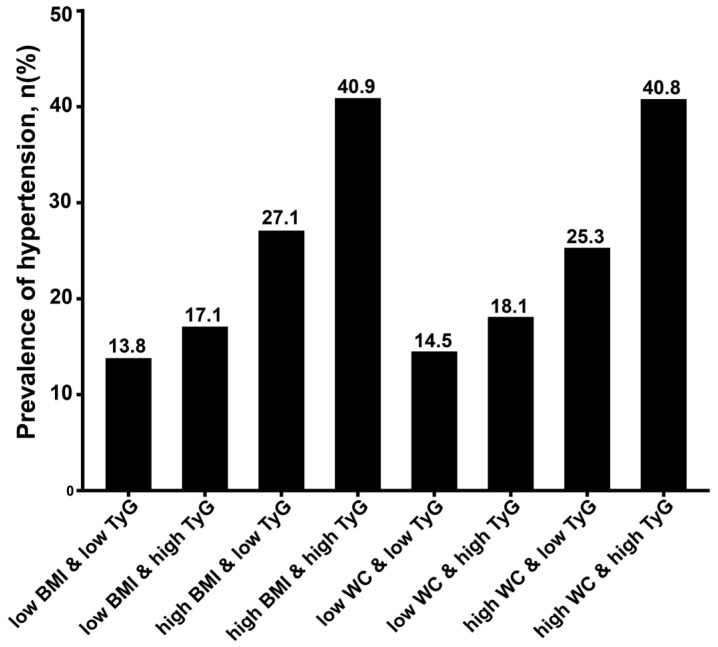
Prevalence of adolescent hypertension across groups of different combinations of obesity and TyG (n = 5703). The cut-off value for defining low/high TyG is its median (8.18), while the cutoffs for low/high obesity indices refer to the sex- and age-specific thresholds set by the Chinese national health industry standards for children and adolescents. TyG, triglyceride-glucose index; BMI, body mass index; WC, waist circumference.

The overall prevalence of adolescent hypertension in five provinces was 19.4%, with regional variations: Guangdong, 11.1%; Jiangsu, 25.1%; Shandong, 23.4%; Guizhou, 20.3%; and Inner Mongolia, 18.4%. Figure 2 illustrates the prevalence of hypertension across groups of different combinations of TyG and obesity indices, and the prevalence reaches approximately 40% when high TyG is combined with high BMI or high WC.

### 3.2. Relationships of TyG, BMI, and WC with Adolescent Hypertension

**Figure 3:**
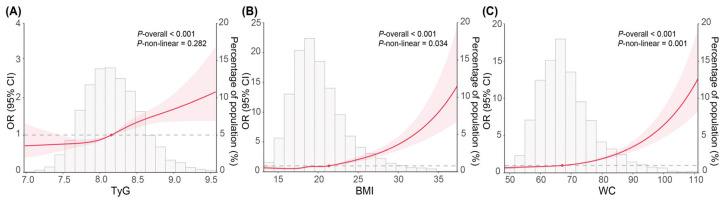
Non-linear relationship of TyG, BMI, and WC with adolescent hypertension, as modeled by restricted cubic spline regression (n = 5703). (**A**) TyG and hypertension; (**B**) BMI and hypertension; (**C**) WC and hypertension. Models were adjusted for sex, age, moderate-vigorous physical activity, unhealthy dietary score, sleep adequacy, secondhand smoke, alcohol intake, family history of hypertension, parental education level, residence, serum uric acid, serum total protein, estimated glomerular filtration rate, total cholesterol, and urine sodium. TyG, triglyceride-glucose index; BMI, body mass index; WC, waist circumference. Shadow shapes indicate 95%CIs.

TyG, BMI, and WC, whether continuous or categorical, were consistently associated with adolescent hypertension prevalence in Model 1 and Model 2 (all *p* < 0.001), as shown in Table 2. After full adjustment (Model 2), the ORs were 1.52 (95%CI: 1.33, 1.75, *p* < 0.001) in high TyG, 3.07 (95%CI: 2.63, 3.58, *p* < 0.001) in high BMI, and 2.71 (95%CI: 2.31, 3.19, *p* < 0.001) in high WC. RCS models (Figure 3) revealed a linear relationship between TyG and hypertension (*p* for non-linear > 0.05), while a non-linear relationship was observed between obesity indices and hypertension (*p* for non-linear < 0.05). Supplementary Materials: Figure S2 shows non-linear relationships of BMI and WC with TyG (*p* for non-linear < 0.001).

### 3.3. Interactive, Joint, and Mediating Effects

Table 3 presents the calculation results of interaction and joint effects. The results suggested that a synergistic interaction exists between IR and obesity on adolescent hypertension. Specifically, significant multiplicative interactions were observed (*p* < 0.05), and SI > 1 further indicated synergistic interactions; RERI > 0 suggested that the joint effect of high TyG and high obesity indices surpassed the sum of their independent effect; and the AP quantified the proportion of excess risk attributable to the combined exposure. In the multivariable logistic regression, the ORs for hypertension were 4.11-fold (95%CI: 3.37, 5.01) and 3.69-fold (95%CI: 3.01, 4.53) higher in the high BMI & high TyG group and the high WC & high TyG group than their corresponding low combined groups. The APs showed that nearly 40% of the hypertension risk associated with concurrent high obesity indices and high TyG in adolescents could be attributed to their interaction.

**Figure 4:**
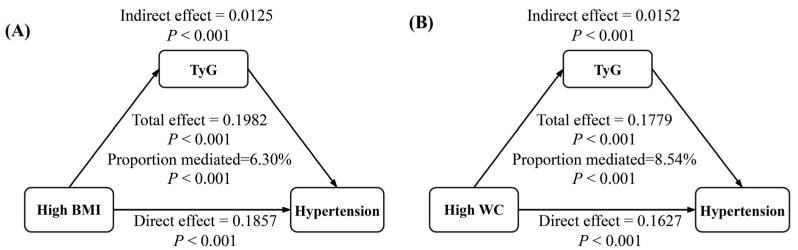
Mediation effects of TyG on the association between obesity and adolescent hypertension (n = 5703). (**A**) Mediation effect of TyG on the high-BMI–hypertension association; (**B**) Mediation effect of TyG on the high-WC–hypertension association. Models were adjusted for sex, age, moderate-vigorous physical activity, unhealthy dietary score, sleep adequacy, secondhand smoke, alcohol intake, family history of hypertension, parental education level, residence, serum uric acid, serum total protein, estimated glomerular filtration rate, total cholesterol, and urine sodium. The cut-off values for low/high obesity indices refer to the sex- and age-specific thresholds set by the Chinese national health industry standards for children and adolescents. TyG, triglyceride-glucose index; BMI, body mass index; WC, waist circumference.

Mediation analysis (Figure 4) demonstrated that TyG significantly mediated the total effect of obesity on adolescent hypertension (*p* < 0.001). The proportions mediated were 6.30% for high BMI and 8.54% for high WC.

### 3.4. Subgroup and Sensitivity Analyses

Table 4 shows that no significant interactions were observed between the stratification variables and the joint effect of TyG and obesity indices on hypertension (*p* for interaction > 0.05), except for age, suggesting that the combined impact of IR and obesity on hypertension varies between individuals aged 12–14 years and 15–17 years. Specifically, adolescents aged 15–17 with high TyG and high obesity indices had greater odds of hypertension than adolescents aged 12–14 (high BMI & high TyG: OR = 3.54 for 12–14 years vs. OR = 5.50 for 15–17 years; high WC & high TyG: OR = 3.17 for 12–14 years vs. OR = 4.85 for 15–17 years). The results of the re-analysis of the interaction in these two age groups show that AP was greater in the adolescents aged 15–17 years than in those aged 12–14 years (Table 5).

Sensitivity analysis revealed that the results using the GLMM modeling approach or using the imputed dataset were consistent with our main analysis, indicating our findings were robust and not affected by province and missing data (Supplementary Materials: Tables S2–S4). The E-value supported the robustness of the association of TyG and obesity indices with adolescent hypertension (Supplementary Materials: Table S5).

## 4. Discussion

Based on a provincially representative surveillance database including five provinces, this study provides a comprehensive perspective regarding the interaction and combined effect of IR and obesity on adolescent hypertension, and the mediating role of IR in the pathway of obesity leading to hypertension in adolescents. The results demonstrated that individuals with both obesity and higher TyG levels had the highest odds of hypertension. Statistically, a synergistic interaction existed between IR and obesity on adolescent hypertension. Moreover, IR mediated the association of obesity with hypertension (Figure 4).

The primary suggestive evidence for pediatric hypertension is currently considered to be excessive weight, salted food intake, and family history of hypertension [3,25]. As a modifiable condition, obesity is a well-recognized risk factor for high blood pressure in both children and adults [26,27]. An umbrella review combined accumulated observational (associative) and genetics-driven (causal) research to gather mutually complementary insights and elucidate the perplexing epidemiological relationship between obesity and cardiovascular disease (including hypertension) [28]. The review found that these two types of studies were concordant, with Mendelian randomization studies supporting adiposity (including general obesity measured by BMI and central obesity measured by WC) as a causal factor for hypertension development. However, not all children with obesity develop hypertension, as some remain normotensive despite excess adiposity [25]. This suggests the need to consider additional metabolic factors when evaluating hypertension risk. As a promising non-insulin-based IR index, a study in 2010 laid the cornerstone for accepting it as a reliable surrogate indicator for evaluating IR [29]. It was reported that TyG showed excellent correlation with the HEC test in diagnosing IR, with an area under the curve (AUC) of 0.858 [29]. Based on simple and low-cost biochemical parameters, TyG is particularly suitable for large-scale epidemiological investigations and resource-limited settings. A substantial body of epidemiological research has demonstrated the link between elevated TyG and the risk of hypertension, including cross-sectional studies and longitudinal cohort studies [8,30,31,32]. Notably, a 2019 study in Mexico was the first to report this association in pediatric populations [30]. Our results on the correlation of TyG with hypertension align with previous literature.

In the development of diseases, especially chronic conditions, the pathogenic factor rarely acts in isolation. Instead, they typically result from a complex interplay of multiple contributing causes. At present, it is thought that obesity-driven fat accumulation leads to hypertension primarily through chronic vascular inflammation, physical compression of the kidney, and overactivation of the sympathetic nervous system (SNS) and the renin–angiotensin–aldosterone system [33,34]. IR contributes to elevating BP through several proposed mechanisms, including SNS activation and increased renal sodium reabsorption [11]. IR can also impair vascular function and structure, promoting the onset and progression of hypertension [33]. Importantly, the accumulation of fat (including visceral fat) can decrease the sensitivity of tissues and cells to insulin, and conversely, IR impairs lipid metabolism, leading to lipid accumulation [33,35]. These intricate pathophysiological networks may underlie the mechanism of the synergistic effects observed between IR and obesity on adolescent hypertension and provide a theoretical foundation for the mediating effects found in our study (Table 3, Figure 4). Although IR may exhibit a physiological increase during puberty, our data emphasize the significance of maintaining relatively low levels of TyG in adolescents. Since obesity can lead to IR, excessive adiposity may transform a physiological increase in IR into a pathological condition. Therefore, weight management is essential for pubertal adolescents.

In the present study, adolescents with a concurrent high obesity index and high TyG had at least a 13.8% higher prevalence of hypertension than those with either factor alone (Figure 2). As expected, our results showed that there were significant biological interactions between the two factors on the prevalence of adolescent hypertension, which epidemiologically confirms the interrelationship between obesity and IR. Our findings are consistent with some studies in adults: IR has a synergistic effect on the obesity–hypertension association among Black and white adults [36]. We also found that the synergistic interaction observed might be influenced by the individuals’ puberty stage, as age subgroups interact with this synergistic effect (*p* for interaction < 0.05) (Table 4). Further analysis showed that the synergistic interaction between obesity and IR was stronger in mid-to-late adolescence (15–17 years) compared with adolescents aged 12–14 years (Table 5). This suggests that adolescents in the age group of 15–17 years who are both obese and have IR may be at a higher risk of developing hypertension.

Given previous research that hypertension prevalence in China is higher in the north than in the south—possibly due to climatic conditions, pollution, or socio-economic factors [37]—we built a GLMM with ‘province’ as a random effect, confirming the robustness of the observed interactions. Actually, we have further tested the effect of province on the interaction term in the main analysis but found no significant heterogeneity (*p* for interaction > 0.05). These results suggest that provinces have no effect on our observation, and given the provincial representativeness of our dataset, this evidence thus supports the generalizability of our results to the national adolescent population.

Between 1990 and 2021, the global prevalence of obesity in children and adolescents tripled [38]. The epidemic of obesity is accompanied by an increasing number of pediatric patients with chronic diseases, including hypertension. However, obesity and IR are both modifiable factors. Healthy lifestyles are helpful to keep IR at an appropriate level, including good sleep, a healthy diet (e.g., more intake of fruits and vegetables, less consumption of added sugars and trans-fats), limiting alcohol, avoiding smoking, and lowering stress through proper means [39]. Furthermore, regular exercise is particularly encouraged because weight loss interventions can significantly lower the risk of hypertension and, importantly, also improve IR, as our findings show [33,39].

Of note, the prevalence of hypertension observed in this study (19.4%) was higher than that reported in most previous studies among children and adolescents [2,3]. However, using the entire national database from this nutrition surveillance, it was found that the prevalence among children and adolescents nationwide in China was 24.9% [40], which is similar to our results. Several factors may account for the relatively high prevalence of hypertension in this surveillance. First, BP was measured using an electronic sphygmomanometer, and prior research has shown that readings obtained with electronic devices tend to be higher than those from mercury sphygmomanometers [41]. In addition, though BP was measured three times at one-minute intervals, it was on a single occasion, which may lead to an overestimation of hypertension prevalence [3,42]. Finally, as mentioned earlier, the growing epidemic of childhood obesity in recent years may have led to dramatic changes in BP levels.

The results in the present study indicated that adolescents with both obesity and IR require particular attention, as obesity and IR might strengthen each other’s association with adolescent hypertension. The combined use of the obesity index and a simple marker of IR could help identify high-risk individuals for hypertension and is beneficial for the prevention and management of hypertension. Future large-scale longitudinal cohort studies will help provide higher-level evidence.

### Limitations

This study has several limitations. First, IR was evaluated by TyG instead of the HEC test, and obesity was estimated by BMI and WC rather than electronic instruments. Studies in the future using more precise measurements could provide more accurate results. Second, considering that our study population consists only of Chinese adolescents and the relationship between IR and BP varies by race [43], it is unclear whether the results of this study can be extended to other ethnicities.

Third, some biases cannot be overlooked. The surveillance data used in this study were collected under strict quality control procedures. A 10% non-response rate was considered during the survey design process. Participants were selected using a standardized sampling method across all survey sites, with strict adherence to randomization principles at each stage of the survey process. Consequently, selection bias was relatively small. All investigators received standardized training before the investigation, and those who did not meet preset criteria underwent additional training to ensure data quality. In addition, certain information regarding participants was collected based on validated structured questionnaires. However, several questionnaire-based covariates may be prone to recall bias, such as secondhand smoke exposure and alcohol use. Single-occasion BP measurement may also lead to misclassification bias. Further, though E-values were calculated to support the robustness of the association of TyG and obesity with adolescent hypertension, residual confounding from unmeasured or unknown factors cannot be excluded, as obesity and IR are intricately related and share many other risk factors.

Fourth, although obesity is a well-established risk factor for hypertension, and IR is thought to be involved in the development of hypertension, the cross-sectional design of this study precludes establishing temporal relationships and thus limits causal inference. Meanwhile, despite some support from the proposed physiological mechanisms, the mediation analysis in this study should be regarded as exploratory due to the constraints of the temporal relationship. Future longitudinal cohort studies are warranted to address this limitation. Finally, it should be noted that it remains unclear whether hypertension in adolescents is a concomitant condition to obesity or obesity is considered as a trigger, or if other interactions between these factors exist. The results of this study should be interpreted with caution.

## 5. Conclusions

This study reveals that IR warrants attention when adolescents are obese, as the two conditions synergistically increase the odds of adolescent hypertension. Our findings indicate that incorporating both obesity and IR into early risk stratification models may help identify adolescents at high risk before the onset of hypertension. Also, strategies simultaneously targeting both obesity and IR should be emphasized for the primary prevention of adolescent hypertension.

## Figures and Tables

**Table 1:** The characteristics of the adolescents from five provinces (n = 5703).

Variables	Hypertension		Total (n = 5703)	*p*-Value
	No (n = 4594)	Yes (n = 1109)		
Age, year	14.2 (1.7)	13.9 (1.7)	14.2 (1.7)	<0.001
Sex, male	2326 (50.63%)	522 (47.07%)	2848 (49.94%)	0.033
BMI, kg/m^2^	19.8 (3.3)	22.0 (4.8)	20.3 (3.7)	<0.001
High BMI (overweight/obesity) *	727 (15.82%)	411 (37.06%)	1138 (19.95%)	<0.001
Waist circumference, cm	68.0 (9.0)	72.3 (12.2)	68.8 (9.8)	<0.001
High WC (abdominal obesity) *	601 (13.08%)	337 (30.39%)	938 (16.45%)	<0.001
MVPA, days/week ^†^	4.0 (2.0, 5.0)	3.0 (2.0, 5.0)	4.0 (2.0, 5.0)	0.006
Unhealthy dietary score	5.9 (1.0)	5.9 (1.0)	5.9 (1.0)	0.156
Adequate sleep	1386 (30.17%)	363 (32.73%)	1749 (30.67%)	0.097
Secondhand smoke				0.462
almost none	3388 (73.75%)	832 (75.02%)	4220 (74.00%)	
1–3 days/week	626 (13.63%)	135 (12.17%)	761 (13.34%)	
4–6 days/week	175 (3.81%)	37 (3.34%)	212 (3.72%)	
everyday	405 (8.82%)	105 (9.47%)	510 (8.94%)	
Alcohol intake				0.002
never	3577 (77.86%)	917 (82.69%)	4494 (78.80%)	
30 days ago	712 (15.50%)	131 (11.81%)	843 (14.78%)	
within 30 days	305 (6.64%)	61 (5.50%)	366 (6.42%)	
Parental education level				0.143
low	1718 (37.40%)	411 (37.06%)	2129 (37.33%)	
medium	2453 (53.40%)	617 (55.64%)	3070 (53.83%)	
high	354 (7.71%)	64 (5.77%)	418 (7.33%)	
unknown	69 (1.50%)	17 (1.53%)	86 (1.51%)	
Family history of hypertension	1599 (34.81%)	381 (34.36%)	1980 (34.72%)	0.008
Rural residence	2042 (44.45%)	459 (41.39%)	2501 (43.85%)	0.065
Province				<0.001
Guangdong	1256 (27.34%)	157 (14.16%)	1413 (24.78%)	
Jiangsu	875 (19.05%)	293 (26.42%)	1168 (20.48%)	
Shandong	1090 (23.73%)	332 (29.94%)	1422 (24.93%)	
Guizhou	598 (13.02%)	152 (13.71%)	750 (13.15%)	
Inner Mongolia	775 (16.87%)	175 (15.78%)	950 (16.66%)	
Urine sodium, mmol/L	144.6 (69.6)	147.2 (71.4)	145.2 (70.0)	0.434
Serum uric acid, μmol/L	363.2 (90.8)	371.3 (96.3)	364.8 (92.0)	0.035
Serum total protein, g/L	76.4 (5.0)	77.7 (5.2)	76.6 (5.1)	<0.001
eGFR, mL/(min·1.73 m^2^)	147.3 (28.6)	147.6 (28.1)	147.4 (28.5)	0.552
TC, mg/dL	149.6 (28.6)	150.8 (30.0)	149.9 (28.9)	0.299
TG, mg/dL	80.8 (33.8)	90.2 (42.0)	82.7 (35.7)	<0.001
FG, mg/dL	93.3 (10.4)	94.8 (11.9)	93.6 (10.7)	<0.001
TyG	8.2 (0.4)	8.3 (0.4)	8.2 (0.4)	<0.001

* The cut-off values for low/high obesity indices refer to the sex- and age-specific thresholds set by the Chinese national health industry standards for children and adolescents. ^†^ MVPA was present as median (interquartile range), other variables were presented as mean (standard deviation) or count (percentage). Abbreviation: BMI, body mass index; WC, waist circumference; MVPA, moderate-vigorous physical activity; eGFR, estimated glomerular filtration rate; TC, total cholesterol; TG, triglyceride; FG, fasting blood glucose; TyG, triglyceride-glucose index.

**Table 2:** Independent association of TyG and obesity indices with hypertension (n = 5703).

Variables *	Model 1		Model 2	
	OR (95%CI)	*p*-Value	OR (95%CI)	*p*-Value
Continuous TyG	1.95 (1.66, 2.31)	<0.001	1.86 (1.56, 2.22)	<0.001
low TyG	Reference		Reference	
high TyG	1.59 (1.40, 1.82)	<0.001	1.52 (1.33, 1.75)	<0.001
Continuous BMI (kg/m^2^)	1.17 (1.15, 1.19)	<0.001	1.16 (1.14, 1.19)	<0.001
low BMI	Reference		Reference	
high BMI	3.22 (2.78, 3.74)	<0.001	3.07 (2.63, 3.58)	<0.001
Continuous WC (cm)	1.05 (1.04, 1.06)	<0.001	1.05 (1.04, 1.06)	<0.001
low WC	Reference		Reference	
high WC	2.94 (2.52, 3.43)	<0.001	2.71 (2.31, 3.19)	<0.001

Model 1 was adjusted for age and sex. Model 2 was adjusted for sex, age, moderate-vigorous physical activity, unhealthy dietary score, sleep adequacy, secondhand smoke, alcohol intake, family history of hypertension, parental education level, residence, serum uric acid, serum total protein, estimated glomerular filtration rate, total cholesterol, and urine sodium. * The cut-off value for defining low/high TyG is its median (8.18), while the cutoffs for low/high obesity indices refer to the sex- and age-specific thresholds set by the Chinese national health industry standards for children and adolescents. Abbreviations: OR, odds ratio; CI, confidence interval; TyG, triglyceride-glucose index; BMI, body mass index; WC, waist circumference.

**Table 3:** Interacting and joint effects of obesity and insulin resistance on adolescent hypertension (n = 5703).

Variables *			OR (95%CI)	*p* for Interaction ^†^	Additive Interaction Measures		
BMI	TyG	WC			RERI (95%CI)	AP (95%CI)	SI (95%CI)
low	low		Reference	0.015	1.60 (0.77, 2.43)	0.39 (0.23, 0.55)	2.06 (1.35, 3.15)
low	high		1.21 (1.03, 1.43)				
high	low		2.30 (1.78, 2.96)				
high	high		4.11 (3.37, 5.01)				
	low	low	Reference	0.007	1.60 (0.79, 2.41)	0.43 (0.26, 0.60)	2.46 (1.42, 4.26)
	high	low	1.23 (1.05, 1.44)				
	low	high	1.86 (1.39, 2.50)				
	high	high	3.69 (3.01, 4.53)				

Models were adjusted for sex, age, moderate-vigorous physical activity, unhealthy dietary score, sleep adequacy, secondhand smoke, alcohol intake, family history of hypertension, parental education level, residence, serum uric acid, serum total protein, estimated glomerular filtration rate, total cholesterol, and urine sodium. * The cut-off value for defining low/high TyG is its median (8.18), while the cutoffs for low/high obesity indices refer to the sex- and age-specific thresholds set by the Chinese national health industry standards for children and adolescents. ^†^ The significance of the multiplicative interaction was evaluated through a likelihood ratio test, comparing models with and without the interaction term. Abbreviations: OR, odds ratio; CI, confidence interval; RERI, relative excess risk due to interaction; AP, proportion attributable to interaction; SI, synergy index; TyG, triglyceride-glucose index; BMI, body mass index; WC, waist circumference.

**Table 4:** Joint effects of obesity and insulin resistance on adolescent hypertension stratified by age, sex, physical activity, and sleep adequacy.

Subgroup (Cases/Total)	Variables *	BMI		WC	
		OR (95%CI) ^†^	*p* for Interaction ^‡^	OR (95%CI) ^†^	*p* for Interaction ^‡^
Age, year			0.019		0.045
12–14 (709/3259)	low BMI/WC & low TyG	Reference		Reference	
	low BMI/WC & high TyG	1.26 (1.02, 1.56)		1.27 (1.04, 1.55)	
	high BMI/WC & low TyG	2.09 (1.50, 2.92)		1.63 (1.10, 2.41)	
	high BMI/WC & high TyG	3.54 (2.75, 4.57)		3.17 (2.44, 4.13)	
15–17 (400/2444)	low BMI/WC & low TyG	Reference		Reference	
	low BMI/WC & high TyG	1.05 (0.79, 1.39)		1.10 (0.84, 1.43)	
	high BMI/WC & low TyG	2.66 (1.78, 3.97)		2.24 (1.45, 3.47)	
	high BMI/WC & high TyG	5.50 (3.98, 7.60)		4.85 (3.49, 6.75)	
Sex			0.806		0.237
male (522/2848)	low BMI/WC & low TyG	Reference		Reference	
	low BMI/WC & high TyG	1.06 (0.82, 1.38)		1.05 (0.83, 1.34)	
	high BMI/WC & low TyG	2.30 (1.64, 3.24)		1.91 (1.27, 2.89)	
	high BMI/WC & high TyG	3.87 (2.94, 5.10)		3.87 (2.91, 5.14)	
female (587/2855)	low BMI/WC & low TyG	Reference		Reference	
	low BMI/WC & high TyG	1.34 (1.07, 1.67)		1.39 (1.12, 1.72)	
	high BMI/WC & low TyG	2.37 (1.60, 3.53)		1.91 (1.26, 2.89)	
	high BMI/WC & high TyG	4.26 (3.17, 5.71)		3.40 (2.51, 4.59)	
MVPA, days/week			0.338		0.360
0–3 (594/2841)	low BMI/WC & low TyG	Reference		Reference	
	low BMI/WC & high TyG	1.40 (1.11, 1.76)		1.41 (1.13, 1.76)	
	high BMI/WC & low TyG	2.49 (1.76, 3.53)		2.11 (1.43, 3.12)	
	high BMI/WC & high TyG	4.05 (3.06, 5.36)		3.70 (2.77, 4.96)	
4–7 (515/2862)	low BMI/WC & low TyG	Reference		Reference	
	low BMI/WC & high TyG	1.03 (0.80, 1.31)		1.04 (0.82, 1.31)	
	high BMI/WC & low TyG	2.09 (1.43, 3.07)		1.62 (1.04, 2.54)	
	high BMI/WC & high TyG	4.05 (3.04, 5.39)		3.61 (2.70, 4.83)	
Adequate sleep			0.065		0.239
no (746/3954)	low BMI/WC & low TyG	Reference		Reference	
	low BMI/WC & high TyG	1.19 (0.97, 1.46)		1.23 (1.01, 1.50)	
	high BMI/WC & low TyG	2.34 (1.72, 3.20)		1.94 (1.36, 2.75)	
	high BMI/WC & high TyG	4.83 (3.80, 6.15)		4.29 (3.35, 5.50)	
yes (363/1749)	low BMI/WC & low TyG	Reference		Reference	
	low BMI/WC & high TyG	1.24 (0.93, 1.66)		1.21 (0.92, 1.60)	
	high BMI/WC & low TyG	2.26 (1.43, 3.57)		1.72 (1.01, 2.94)	
	high BMI/WC & high TyG	2.90 (2.03, 4.14)		2.60 (1.86, 3.87)	

* The cut-off value for defining low/high TyG is its median (8.18), while the cutoffs for low/high obesity indices refer to the sex- and age-specific thresholds set by the Chinese national health industry standards for children and adolescents. ^†^ Models were adjusted for, if not stratified, sex, age, moderate-vigorous physical activity, unhealthy dietary score, sleep adequacy, secondhand smoke, alcohol intake, family history of hypertension, parental education, residence, serum uric acid, serum total protein, estimated glomerular filtration rate, total cholesterol, and urine sodium. ^‡^ *p* for interaction was derived from logistic regression models by comparing models including and not including the stratification variable in the interaction terms (TyG with BMI or WC) to test for heterogeneity of the studied interactive effects across subgroups. Abbreviations: OR, odds ratio; CI, confidence interval; TyG, triglyceride-glucose index; BMI, body mass index; WC, waist circumference; MVPA, moderate-vigorous physical activity.

**Table 5:** Interactive effects of obesity and insulin resistance on hypertension in adolescents aged 12–14 years and 15–17 years.

Interactive Items *	Measures ^†^	12–14 Years Old (n = 3259)		15–17 Years Old (n = 2444)	
		Coefficients (95%CI)	*p*-Value	Coefficients (95%CI)	*p*-Value
BMI and TyG	Additive effects				
	RERI	1.18 (0.25, 2.12)	0.007	2.79 (1.03, 4.55)	0.001
	AP	0.33 (0.11, 0.56)	0.002	0.51 (0.29, 0.73)	<0.001
	SI	1.87 (1.07, 3.29)	0.014	2.63 (1.35, 5.14)	0.002
WC and TyG	Additive effects				
	RERI	1.28 (0.36, 2.19)	0.003	2.51 (0.87, 4.16)	0.002
	AP	0.40 (0.17, 0.64)	<0.001	0.52 (0.28, 0.75)	<0.001
	SI	2.42 (1.10, 5.33)	0.014	2.88 (1.30, 6.39)	0.005

* Additive interaction measures were calculated based on the reference group with low TyG & low BMI or low WC. The cut-off value for defining low/high TyG is its median (8.18), while the cutoffs for low/high obesity indices refer to the sex- and age-specific thresholds set by the Chinese national health industry standards for children and adolescents. ^†^ Models were adjusted for sex, moderate-vigorous physical activity, unhealthy dietary score, sleep adequacy, secondhand smoke, alcohol intake, family history of hypertension, parental education level, residence, serum uric acid, serum total protein, estimated glomerular filtration rate, total cholesterol, and urine sodium. Abbreviations: CI, confidence interval; RERI, relative excess risk due to interaction; AP, proportion attributable to interaction; SI, synergy index; TyG, triglyceride-glucose index; BMI, body mass index; WC, waist circumference.

## Data Availability

The datasets used and/or analyzed during the current study are not publicly available due to ethical and privacy reasons, but are available from the corresponding author upon reasonable request.

## Supplementary Materials

The following supporting information can be downloaded at: https://www.mdpi.com/article/10.3390/nu17172783/s1, Figure S1. Missing variables in our original dataset of 7031 adolescents aged 12–17 years old. Figure S2. Non-linear relationship of body mass index and waist circumference with triglyceride-glucose index (n = 5703). Table S1. Baseline characteristics between adolescents included and not included. Table S2. Interacting and joint effects of obesity and insulin resistance on adolescent hypertension based on generalized linear mixed-effect models. Table S3. The characteristics of the adolescents from five provinces after multiple imputation. Table S4. Interacting and joint effects of obesity and insulin resistance on adolescent hypertension in samples after multiple imputation. Table S5. E-values of high TyG, high BMI, and high WC with adolescent hypertension.

## Abbreviations

TyG, triglyceride-glucose index; BMI, body mass index; WC, waist circumference; BP, blood pressure; MVPA, moderate-vigorous physical activity; eGFR, estimated glomerular filtration rate; TC, total cholesterol; TG, triglyceride; FG, fasting blood glucose; OR, odds ratio; CI, confidence interval; RERI, relative excess risk due to interaction; AP, proportion attributable to interaction; SI, synergy index; AUC, area under the curve.

## References

[1] Robinson C.H., Chanchlani R. (2022). High Blood Pressure in Children and Adolescents: Current Perspectives and Strategies to Improve Future Kidney and Cardiovascular Health. Kidney Int. Rep..

[2] Song P., Zhang Y., Yu J., Zha M., Zhu Y., Rahimi K., Rudan I. (2019). Global Prevalence of Hypertension in Children: A Systematic Review and Meta-Analysis. JAMA Pediatr..

[3] Zhou J., Wu J., Jiang D., Cai S., Zhang C., Ying J., Cao J., Song Y., Song P. (2024). National, Regional and Provincial Prevalence of Childhood Hypertension in China in 2020: A Systematic Review and Modelling Study. Lancet Child Adolesc. Health.

[4] Yang L., Magnussen C.G., Yang L., Bovet P., Xi B. (2020). Elevated Blood Pressure in Childhood or Adolescence and Cardiovascular Outcomes in Adulthood: A Systematic Review. Hypertension.

[5] Shankar R.R., Eckert G.J., Saha C., Tu W., Pratt J.H. (2005). The Change in Blood Pressure during Pubertal Growth. J. Clin. Endocrinol. Metab..

[6] Ewald D.R., Haldeman L.A. (2016). Risk Factors in Adolescent Hypertension. Glob. Pediatr. Health.

[7] Din-Dzietham R., Liu Y., Bielo M.-V., Shamsa F. (2007). High Blood Pressure Trends in Children and Adolescents in National Surveys, 1963 to 2002. Circulation.

[8] Tsai K.-Z., Chu C.-C., Huang W.-C., Sui X., Lavie C.J., Lin G.-M. (2024). Prediction of Various Insulin Resistance Indices for the Risk of Hypertension among Military Young Adults: The CHIEF Cohort Study, 2014–2020. Cardiovasc. Diabetol..

[9] Zhao X., An X., Yang C., Sun W., Ji H., Lian F. (2023). The Crucial Role and Mechanism of Insulin Resistance in Metabolic Disease. Front. Endocrinol..

[10] Wang K., He G., Zhang Y., Yin J., Yan Y., Zhang Y., Wang K. (2021). Association of Triglyceride-Glucose Index and Its Interaction with Obesity on Hypertension Risk in Chinese: A Population-Based Study. J. Hum. Hypertens..

[11] Chen Y., Hu P., He Y., Qin H., Hu L., Yang R. (2024). Association of TyG Index and Central Obesity with Hypertension in Middle-Aged and Elderly Chinese Adults: A Prospective Cohort Study. Sci. Rep..

[12] Kang B., Yang Y., Lee E.Y., Yang H.K., Kim H.-S., Lim S.-Y., Lee J.-H., Lee S.-S., Suh B.-K., Yoon K.-H. (2017). Triglycerides/Glucose Index Is a Useful Surrogate Marker of Insulin Resistance among Adolescents. Int. J. Obes..

[13] Calcaterra V., Verduci E., Vandoni M., Rossi V., Fiore G., Massini G., Berardo C., Gatti A., Baldassarre P., Bianchi A. (2022). The Effect of Healthy Lifestyle Strategies on the Management of Insulin Resistance in Children and Adolescents with Obesity: A Narrative Review. Nutrients.

[14] Yazıcı D., Sezer H. (2017). Insulin Resistance, Obesity and Lipotoxicity. Adv. Exp. Med. Biol..

[15] Yu D., Zhao L., Zhang J., Yang Z., Yang L., Huang J., Fang H., Guo Q., Xu X., Ju L. (2021). China Nutrition and Health Surveys (1982−2017). China CDC Wkly..

[16] Anthropometric Measurements Method in Health Surveillance. WS/T 424—2013. https://www.nhc.gov.cn/wjw/yingyang/201308/1f27caef0b22493e93a1da8aec2cd63a/files/1739783545878_39188.pdf.

[17] Screening for Overweight and Obesity Among School-Age Children and Adolescents. WS/T 586—2018. http://www.nhc.gov.cn/ewebeditor/uploadfile/2018/03/20180330094031236.pdf.

[18] Cheng X., Guo Q., Ju L., Gong W., Wei X., Xu X., Zhao L., Fang H. (2024). Association between Sedentary Behavior, Screen Time and Metabolic Syndrome among Chinese Children and Adolescents. BMC Public Health.

[19] High Waist Circumference Screening Threshold Among Children and Adolescents Aged 7−18 Years. WS/T 611—2018. http://www.nhc.gov.cn/ewebeditor/uploadfile/2018/07/20180704145130574.pdf.

[20] Reference of Screening for Elevated Blood Pressure Among Children and Adolescents Aged 7−18 Years. WS/T 610—2018. http://www.nhc.gov.cn/ewebeditor/uploadfile/2018/07/20180705095101600.pdf.

[21] The State Council of the People’s Republic of China China Sets Minimum Sleeping Hours for Children, Teens. https://english.www.gov.cn/statecouncil/ministries/202104/03/content_WS6067ac56c6d0719374afc027.html?utm_source=chatgpt.com.

[22] The Chinese Nutrition Society The Chinese Dietary Guidelines. http://dg.cnsoc.org/imgnewslist_0602_2.htm.

[23] Zhu H., Wu Q., Zhang R., Zhang Z., Feng Y., Liu T., Liu D., Chen X., Dong X. (2025). Protective Association of Weekend Catch-up Sleep with Metabolic Syndrome in Chinese Children and Adolescents with Sleep Insufficiency. Sleep Med..

[24] Subspecialty Group of Nephrology, the Society of Pediatrics, Chinese Medical Association, Editorial Board, Chinese Journal of Pediatrics (2022). Clinical practice guideline for early screening of pediatric chronic kidney disease in China (version 2021). Chin. J. Pediatr..

[25] Astudillo Y., Kibrom S., Pereira T., Solomon S., Krishnan S., Samsonov D. (2024). Association between Anxiety and Elevated Blood Pressure in Adolescent Patients: A Single-Center Cross-Sectional Study. J. Hypertens..

[26] Rutigliano I., De Filippo G., Pastore L., Messina G., Agostoni C., Campanozzi A. (2021). Obesity-Related Hypertension in Pediatrics, the Impact of American Academy of Pediatrics Guidelines. Nutrients.

[27] Roush G.C. (2019). Obesity-Induced Hypertension: Heavy on the Accelerator. J. Am. Heart Assoc. Cardiovasc. Cerebrovasc. Dis..

[28] Kim M.S., Kim W.J., Khera A.V., Kim J.Y., Yon D.K., Lee S.W., Shin J.I., Won H.-H. (2021). Association between Adiposity and Cardiovascular Outcomes: An Umbrella Review and Meta-Analysis of Observational and Mendelian Randomization Studies. Eur. Heart J..

[29] Guerrero-Romero F., Simental-Mendía L.E., González-Ortiz M., Martínez-Abundis E., Ramos-Zavala M.G., Hernández-González S.O., Jacques-Camarena O., Rodríguez-Morán M. (2010). The Product of Triglycerides and Glucose, a Simple Measure of Insulin Sensitivity. Comparison with the Euglycemic-Hyperinsulinemic Clamp. J. Clin. Endocrinol. Metab..

[30] Simental-Mendía L.E., Hernández-Ronquillo G., Gamboa-Gómez C.I., Gómez-Díaz R., Rodríguez-Morán M., Guerrero-Romero F. (2019). The Triglycerides and Glucose Index Is Associated with Elevated Blood Pressure in Apparently Healthy Children and Adolescents. Eur. J. Pediatr..

[31] Xu J., Xu W., Chen G., Hu Q., Jiang J. (2023). Association of TyG Index with Prehypertension or Hypertension: A Retrospective Study in Japanese Normoglycemia Subjects. Front. Endocrinol..

[32] Xin F., He S., Zhou Y., Jia X., Zhao Y., Zhao H. (2023). The Triglyceride Glucose Index Trajectory Is Associated with Hypertension: A Retrospective Longitudinal Cohort Study. Cardiovasc. Diabetol..

[33] Parvanova A., Reseghetti E., Abbate M., Ruggenenti P. (2023). Mechanisms and Treatment of Obesity-Related Hypertension—Part 1: Mechanisms. Clin. Kidney J..

[34] Dorresteijn J.A.N., Visseren F.L.J., Spiering W. (2012). Mechanisms Linking Obesity to Hypertension. Obes. Rev. Off. J. Int. Assoc. Study Obes..

[35] Lin M., Chen X., Wu M., Xiao J., Li S., Tang H., Tan X., Chen Y. (2025). Interactive Effects of Abdominal Obesity and Insulin Resistance on Cardiometabolic Risk. Rev. Espanola Cardiol. Engl. Ed..

[36] Zhang T., Zhang H., Li S., Li Y., Liu Y., Fernandez C., Harville E., Bazzano L., He J., Chen W. (2016). Impact of Adiposity on Incident Hypertension Is Modified by Insulin Resistance in Adults: Longitudinal Observation from the Bogalusa Heart Study. Hypertension.

[37] Li Y., Wang L., Feng X., Zhang M., Huang Z., Deng Q., Zhou M., Astell-Burt T., Wang L. (2018). Geographical Variations in Hypertension Prevalence, Awareness, Treatment and Control in China: Findings from a Nationwide and Provincially Representative Survey. J. Hypertens..

[38] Kerr J.A., Patton G.C., Cini K.I., Abate Y.H., Abbas N., Magied A.H.A.A.A., ElHafeez S.A., Abd-Elsalam S., Abdollahi A., Abdoun M. (2025). Global, Regional, and National Prevalence of Child and Adolescent Overweight and Obesity, 1990–2021, with Forecasts to 2050: A Forecasting Study for the Global Burden of Disease Study 2021. Lancet.

[39] Li M., Chi X., Wang Y., Setrerrahmane S., Xie W., Xu H. (2022). Trends in Insulin Resistance: Insights into Mechanisms and Therapeutic Strategy. Signal Transduct. Target. Ther..

[40] Yang Y., Li Y., Yuan H., Tang Z., Chen M., Cai S., Piao W., Nan J., Li F., Yu D. (2024). Hypertension-Related Status and Influencing Factors among Chinese Children and Adolescents Aged 6~17 Years: Data from China Nutrition and Health Surveillance (2015–2017). Nutrients.

[41] Cao Q., Li D., Yu W., Guo Q., Zhao L., Yu D., Wang Y. (2016). Consistency between the electronic and mercury sphygmomanometer in children and adolescents on blood pressure measuring. Wei Sheng Yan Jiu.

[42] Dong J., Dong H., Yan Y., Cheng H., Zhao X., Mi J. (2022). Prevalence of Hypertension and Hypertension Phenotypes after Three Visits in Chinese Urban Children. J. Hypertens..

[43] Saad M.F., Lillioja S., Nyomba B.L., Castillo C., Ferraro R., De Gregorio M., Ravussin E., Knowler W.C., Bennett P.H., Howard B.V. (1991). Racial Differences in the Relation between Blood Pressure and Insulin Resistance. N. Engl. J. Med..

