# Supplementary material for: Interactive and Joint Effects of Obesity and Insulin Resistance on Hypertension in Adolescents and the Mediating Role of Insulin Resistance—Five Provinces, China
Source: Nutrients. 2025 Aug 27;17(17):2783. doi: 10.3390/nu17172783 (PMC12430777; doi:10.3390/nu17172783)
Supplement: Supplementary file 1 [file nutrients-17-02783-s001.zip › nutrients-3799172-supplementary.pdf]

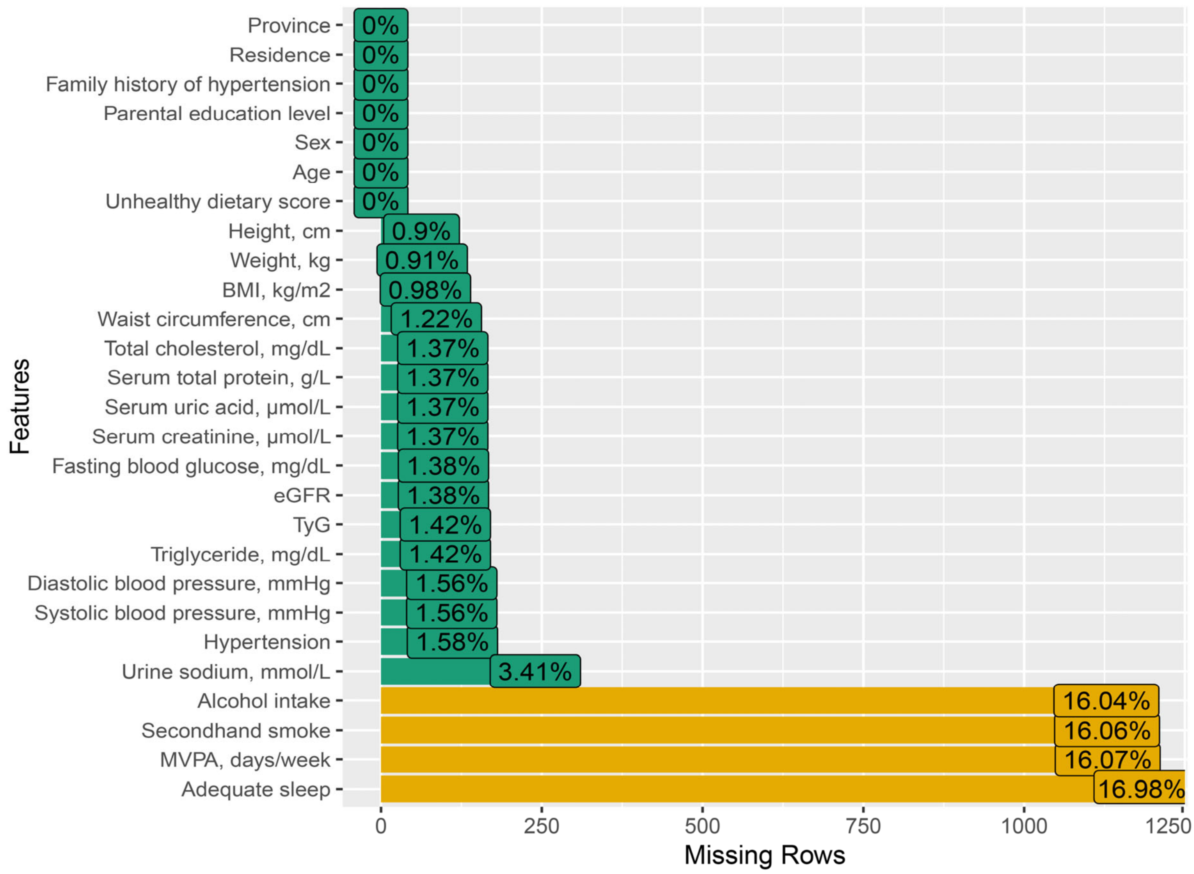

Figure S1. Missing variables in our original dataset of 7031 adolescents aged 12–17 years old. Note: The number of missing variables shown in the exclusion flowchart (Figure 1) in the main text changes throughout the exclusion process and therefore does not completely align with the missingness presented in Figure S1.

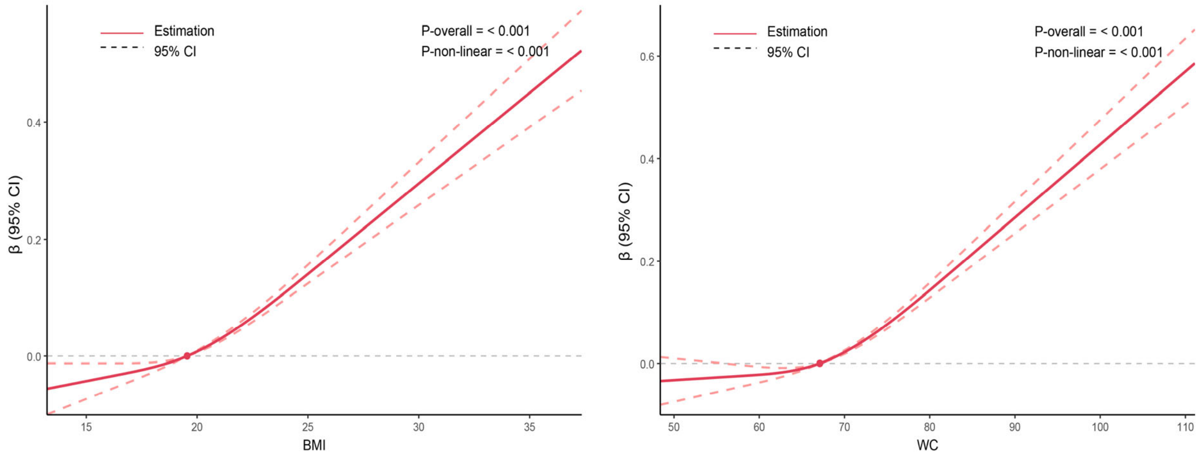

Figure S2. Non-linear relationship of body mass index and waist circumference with triglyceride-glucose index (n=5703). Models were adjusted for sex, age, moderate-vigorous physical activity, unhealthy dietary score, sleep adequacy, secondhand smoke, alcohol intake, family history of hypertension, parental education level, residence, serum uric acid, serum total protein, estimated glomerular filtration rate, total cholesterol, and urine sodium. CI, confidence interval; BMI, body mass index; WC, waist circumference.

Table S1. Baseline characteristics between adolescents included and not included.

Variables	Include (n=5703)	Exclude (n=1328)	*P*-value*
Age, mean (SD), year	14.2 (1.7)	14.3 (1.7)	0.016
missing count	0	0	
Sex, male	2848 (49.94%)	685 (51.58%)	0.281
missing count	0	0	
BMI, mean (SD), kg/m^2	20.3 (3.7)	20.1 (3.6)	0.070
missing count	0	69	
Waist circumference, mean (SD), cm	68.8 (9.8)	68.2 (10.0)	0.068
missing count	0	86	
Hypertension	1109 (19.45%)	210 (17.26%)	0.077
missing count	0	111	
MVPA, median (IQR), days/week	4.0 (2.0, 5.0)	2.0 (1.5, 5.0)	0.003
missing count	0	1129	
Unhealthy dietary score, mean (SD)	5.9 (1.0)	6.0 (0.9)	<0.001
missing count	0	0	
Adequate sleep	1749 (30.67%)	35 (26.12%)	0.259
missing count	0	1194	
Secondhand smoke			0.460
almost none	4220 (74.00%)	138 (69.35%)	
1–3 days/week	761 (13.34%)	32 (16.08%)	
4–6 days/week	212 (3.72%)	10 (5.03%)	
everyday	510 (8.94%)	19 (9.55%)	
missing count	0	1129	
Alcohol intake			0.888
never	4494 (78.80%)	155 (77.50%)	
more than 30 days ago	843 (14.78%)	32 (16.00%)	
in the last 30 days	366 (6.42%)	13 (6.50%)	
missing count	0	1128	
Parental education level			0.005
low	2129 (37.33%)	555 (41.79%)	
medium	3070 (53.83%)	647 (48.72%)	
high	418 (7.33%)	110 (8.28%)	
unknown	86 (1.51%)	16 (1.20%)	
missing count	0	0	
Family history of hypertension	1980 (34.72%)	59 (4.44%)	<0.001
missing count	0	0	
Rural residence	2501 (43.85%)	680 (51.20%)	<0.001

missing count	0	0	
Province			<0.001
Guangdong	1413 (24.78%)	211 (15.89%)	
Jiangsu	1168 (20.48%)	272 (20.48%)	
Shandong	1422 (24.93%)	276 (20.78%)	
Guizhou	750 (13.15%)	529 (39.83%)	
Inner Mongolia	950 (16.66%)	40 (3.01%)	
missing count	0	0	
Urine sodium, mean (SD), mmol/L	145.2 (70.0)	151.4 (74.1)	0.012
missing count	0	104	
Serum uric acid, mean (SD), μmol/L	364.8 (92.0)	362.1 (93.1)	0.296
missing count	0	96	
Serum total protein, mean (SD), g/L	76.6 (5.1)	77.2 (5.2)	<0.001
missing count	0	96	
eGFR, mean (SD), ml/(min·1.73m^2)	147.4 (28.5)	146.8 (27.4)	0.768
missing count	0	98	
Total cholesterol, mean (SD), mg/dL	149.9 (28.9)	146.4 (28.2)	<0.001
missing count	0	96	
Triglyceride, mean (SD), mg/dL	82.7 (35.7)	85.8 (37.0)	0.005
missing count	0	100	
Fasting blood glucose, mean (SD), mg/dL	93.6 (10.7)	92.0 (11.2)	<0.001
missing count	0	97	
TyG, mean (SD)	8.2 (0.4)	8.2 (0.4)	0.170
missing count	0	100	

Variables were presented as mean (standard deviation) or count (percentage).

**P*-value was based on Pearson's chi-square test, Student's t test, or Wilcoxon rank sum test, where appropriate.

Abbreviation: SD, standard deviation; IQR, interquartile range; BMI, body mass index; MVPA, moderate-vigorous physical activity; eGFR, estimated glomerular filtration rate; TyG, triglyceride-glucose index.

Table S2. Interacting and joint effects of obesity and insulin resistance on adolescent hypertension based on generalized linear mixed-effect models.

Variables*			OR (95%CI)	*P* for interaction†	Additive interaction measures		
BMI	TyG	WC			RERI (95% CI)	AP (95% CI)	SI (95% CI)
low	low		Reference	0.036	1.27 (0.90, 1.64)	0.35 (0.25, 0.46)	1.96 (1.68, 2.24)
low	high		1.21 (1.03, 1.43)				
high	low		2.11 (1.63, 2.73)				
high	high		3.59 (2.93, 4.40)				
	low	low	Reference	0.024	1.22 (0.82, 1.61)	0.39 (0.26, 0.51)	2.29 (1.87, 2.71)
	high	low	1.23 (1.05, 1.45)				
	low	high	1.71 (1.27, 2.29)				
	high	high	3.16 (2.56, 3.90)				

Models were adjusted for sex, age, moderate-vigorous physical activity, unhealthy dietary score, sleep adequacy, secondhand smoke, alcohol intake, family history of hypertension, parental education level, residence, serum uric acid, serum total protein, estimated glomerular filtration rate, total cholesterol, and urine sodium.

*The cut-off value for defining low/high TyG is its median (8.18), while the cutoffs for low/high obesity indices refer to the sex- and age-specific thresholds set by the Chinese national health industry standards for children and adolescents.

†The significance of the multiplicative interaction was evaluated through a likelihood ratio test, comparing models with and without the interaction term.

Abbreviations: OR, odds ratio; CI, confidence interval; RERI, relative excess risk due to interaction; AP, proportion attributable to interaction; SI, synergy index; TyG, triglyceride-glucose index; BMI, body mass index; WC, waist circumference.

Table S3. The characteristics of the adolescents from five provinces after multiple imputation.

Variables	Hypertension		Total (n=7031)	*P*-value*
	no (n=5693)	yes (n=1338)		
Age, year	14.2 (1.7)	14.0 (1.7)	14.2 (1.7)	<0.001
Sex, male	2900 (50.94%)	633 (47.31%)	3533 (50.25%)	0.033
High BMI (overweight/obesity)†	807 (14.18%)	462 (34.53%)	1269 (18.05%)	<0.001
High WC (abdominal obesity)†	720 (12.65%)	408 (30.49%)	1128 (16.04%)	<0.001
MVPA, days/week				<0.001
0–3	2337 (41.05%)	627 (46.86%)	2964 (42.16%)	
4–7	2411 (42.35%)	527 (39.39%)	2938 (41.79%)	
unknown	945 (16.60%)	184 (13.75%)	1129 (16.06%)	
Unhealthy dietary score	5.9 (1.0)	5.9 (1.0)	5.9 (1.0)	0.284
Adequate sleep				0.008
no	3291 (57.81%)	762 (56.95%)	4053 (57.64%)	
yes	1407 (24.71%)	377 (28.18%)	1784 (25.37%)	
unknown	995 (17.48%)	199 (14.87%)	1194 (16.98%)	
Secondhand smoke				0.062
almost none	3496 (61.41%)	862 (64.42%)	4358 (61.98%)	
1–3 days/week	650 (11.42%)	143 (10.69%)	793 (11.28%)	
4–6 days/week	183 (3.21%)	39 (2.91%)	222 (3.16%)	
everyday	419 (7.36%)	110 (8.22%)	529 (7.52%)	
unknown	945 (16.60%)	184 (13.75%)	1129 (16.06%)	
Alcohol intake				<0.001
never	3696 (64.92%)	953 (71.23%)	4649 (66.12%)	
30 days ago	737 (12.95%)	138 (10.31%)	875 (12.44%)	
within 30 days	315 (5.53%)	64 (4.78%)	379 (5.39%)	
unknown	945 (16.60%)	183 (13.68%)	1128 (16.04%)	
Parental education level				0.036
low	2175 (38.20%)	509 (38.04%)	2684 (38.17%)	
medium	2984 (52.42%)	733 (54.78%)	3717 (52.87%)	
high	452 (7.94%)	76 (5.68%)	528 (7.51%)	
unknown	82 (1.44%)	20 (1.49%)	102 (1.45%)	
Family history of hypertension	1642 (28.84%)	397 (29.67%)	2039 (29.00%)	<0.001
Rural residence	2609 (45.83%)	572 (42.75%)	3181 (45.24%)	0.042
Urine sodium, mmol/L	145.8 (70.3)	148.5 (72.5)	146.3 (70.7)	0.422
Serum uric acid, μmol/L	362.7 (90.9)	371.3 (97.1)	364.4 (92.2)	0.015
Serum total protein, g/L	76.5 (5.1)	77.7 (5.2)	76.7 (5.1)	<0.001
eGFR, ml/(min·1.73m^2)	147.3 (28.6)	147.3 (28.3)	147.3 (28.6)	0.740

TC, mg/dL	149.0 (28.7)	150.5 (29.5)	149.3 (28.9)	0.101
TyG	8.2 (0.4)	8.3 (0.4)	8.2 (0.4)	<0.001

Variables were presented as mean (standard deviation) or count (percentage).

[*] *P*-value was based on Pearson's chi-square test, Student's t test, or Wilcoxon rank sum test, where appropriate.

[†] The cut-off values for low/high obesity indices refer to the sex- and age-specific thresholds set by the Chinese national health industry standards for children and adolescents.

Abbreviation: BMI, body mass index; WC, waist circumference; MVPA, moderate-vigorous physical activity; eGFR, estimated glomerular filtration rate; TC, total cholesterol; TyG, triglyceride-glucose index.

Table S4. Interacting and joint effects of obesity and insulin resistance on adolescent hypertension in samples after multiple imputation.

Variables*			OR (95%CI)	*P* for interaction†	Additive interaction measures		
BMI	TyG	WC			RERI (95% CI)	AP (95% CI)	SI (95% CI)
low	low		Reference	0.041	1.52 (0.69 2.34)	0.36 (0.20, 0.52)	1.87 (1.29, 2.73)
low	high		1.27 (1.09, 1.47)				
high	low		2.47 (1.94, 3.14)				
high	high		4.25 (3.54, 5.11)				
	low	low	Reference	0.033	1.43 (0.64, 2.22)	0.37 (0.20, 0.54)	2.01 (1.29, 3.12)
	high	low	1.27 (1.10, 1.47)				
	low	high	2.14 (1.65, 2.79)				
	high	high	3.84 (3.19, 4.63)				

Models were adjusted for sex, age, moderate-vigorous physical activity, unhealthy dietary score, sleep adequacy, secondhand smoke, alcohol intake, family history of hypertension, parental education level, residence, serum uric acid, serum total protein, estimated glomerular filtration rate, total cholesterol, and urine sodium.

*The cut-off value for defining low/high TyG is its median (8.18), while the cutoffs for low/high obesity indices refer to the sex- and age-specific thresholds set by the Chinese national health industry standards for children and adolescents.

†The significance of the multiplicative interaction was evaluated through a likelihood ratio test, comparing models with and without the interaction term.

Abbreviations: OR, odds ratio; CI, confidence interval; RERI, relative excess risk due to interaction; AP, proportion attributable to interaction; SI, synergy index; TyG, triglyceride-glucose index; BMI, body mass index; WC, waist circumference.

Table S5. E-values of high TyG, high BMI, and high WC with adolescent hypertension.

Variables*	E-value (95% CI)
TyG	
low TyG	Reference
high TyG	1.60 (1.39)
BMI	
low BMI	Reference
high BMI	2.82 (2.55)
WC	
low WC	Reference
high WC	2.58 (2.31)

Models were adjusted for sex, age, moderate-vigorous physical activity, unhealthy dietary score, sleep adequacy, secondhand smoke, alcohol intake, family history of hypertension, parental education level, residence, serum uric acid, serum total protein, estimated glomerular filtration rate, total cholesterol, and urine sodium.

*The cut-off value for defining low/high TyG is its median (8.18), while the cutoffs for low/high obesity indices refer to the sex- and age-specific thresholds set by the Chinese national health industry standards for children and adolescents.

Abbreviations: CI, confidence interval; TyG, triglyceride-glucose index; BMI, body mass index; WC, waist circumference.
